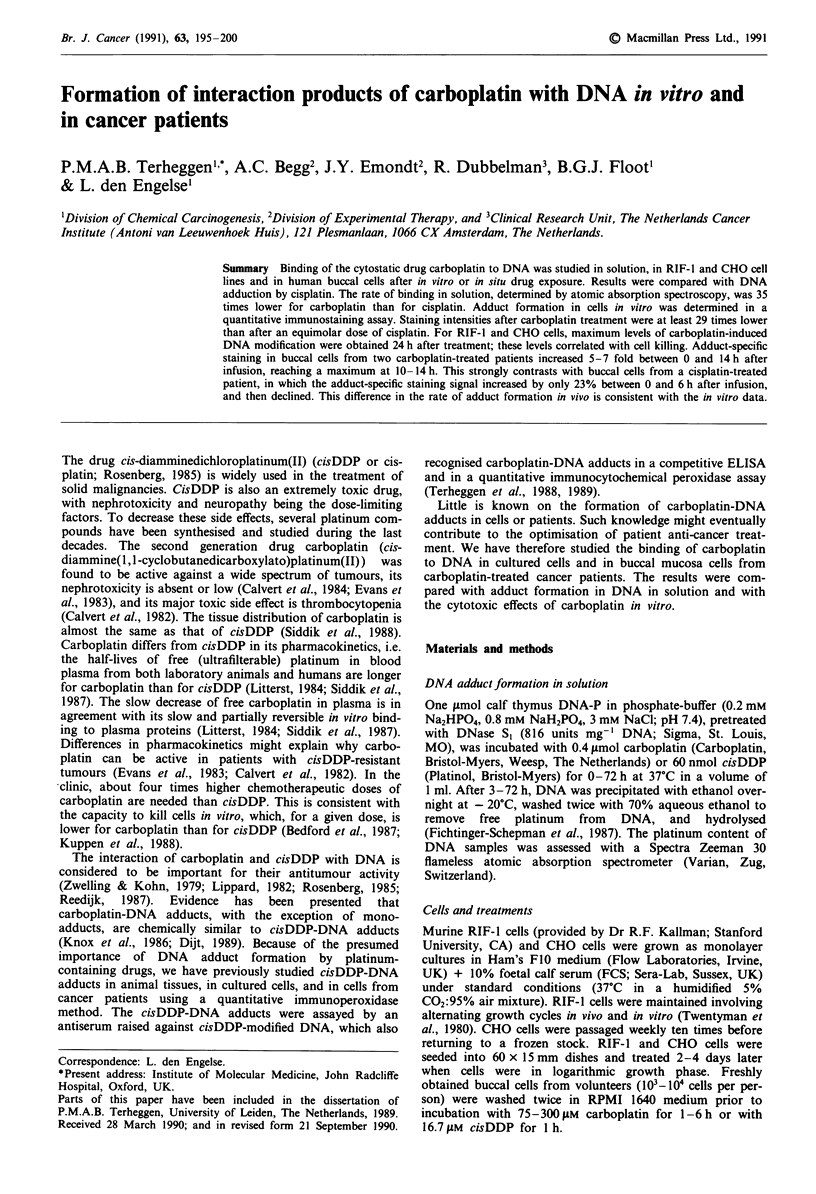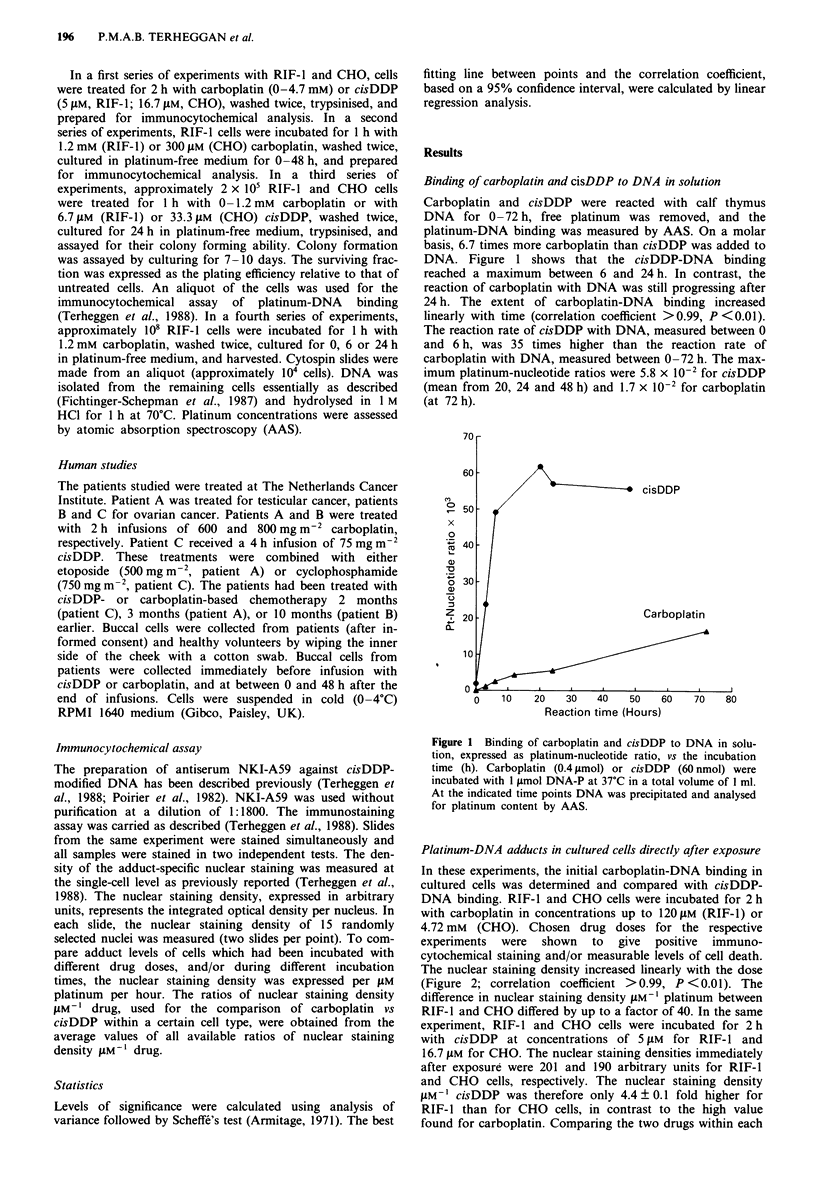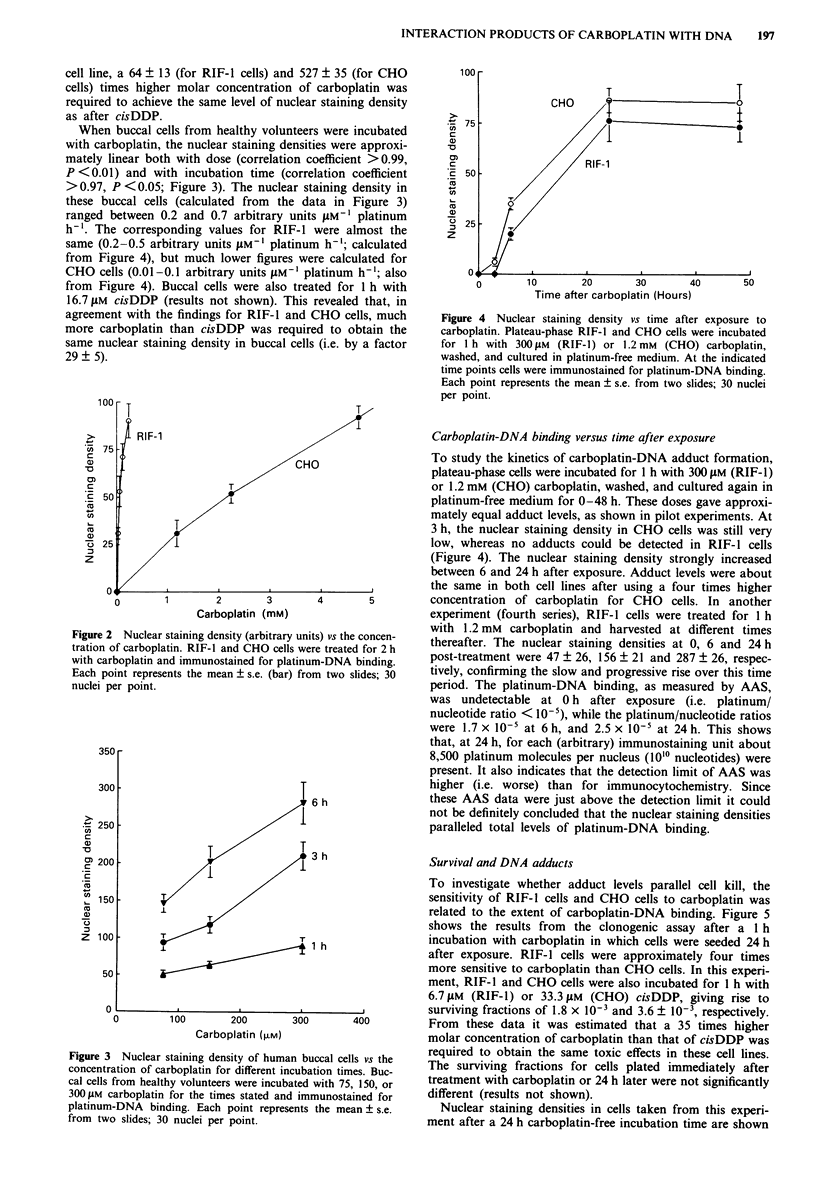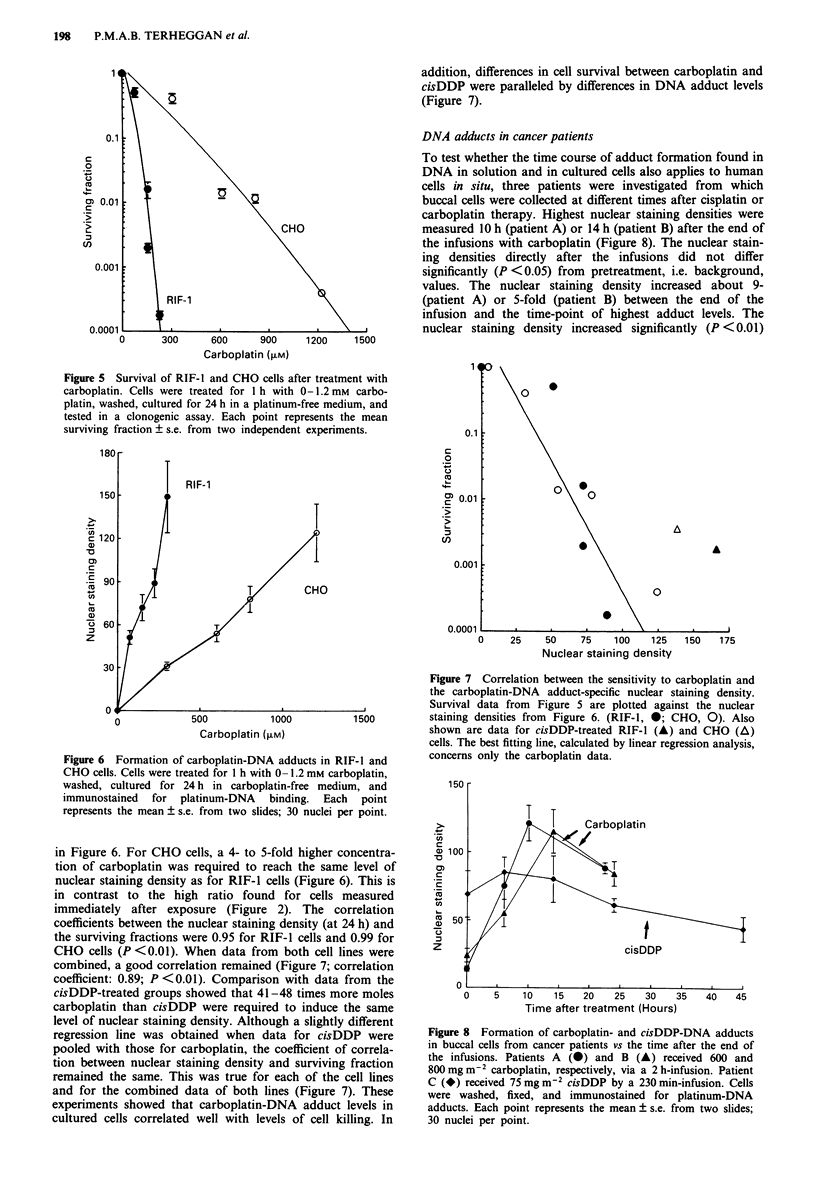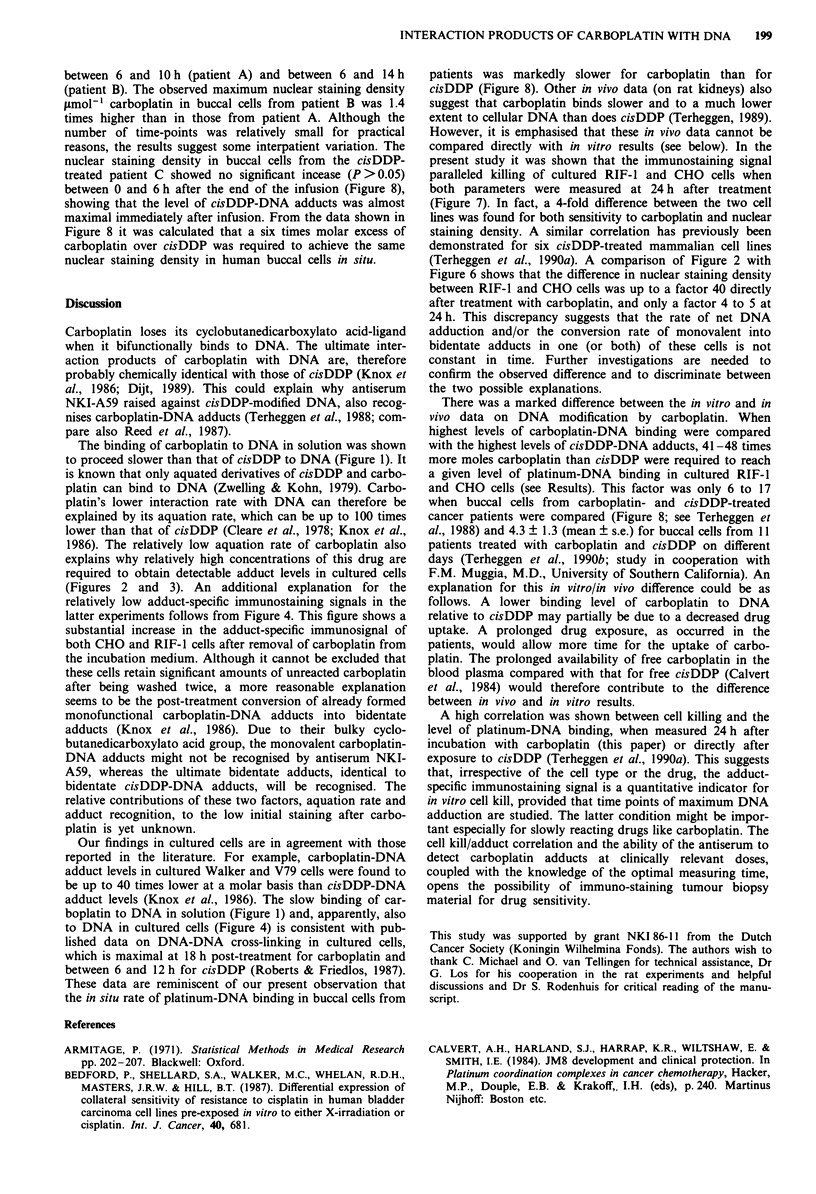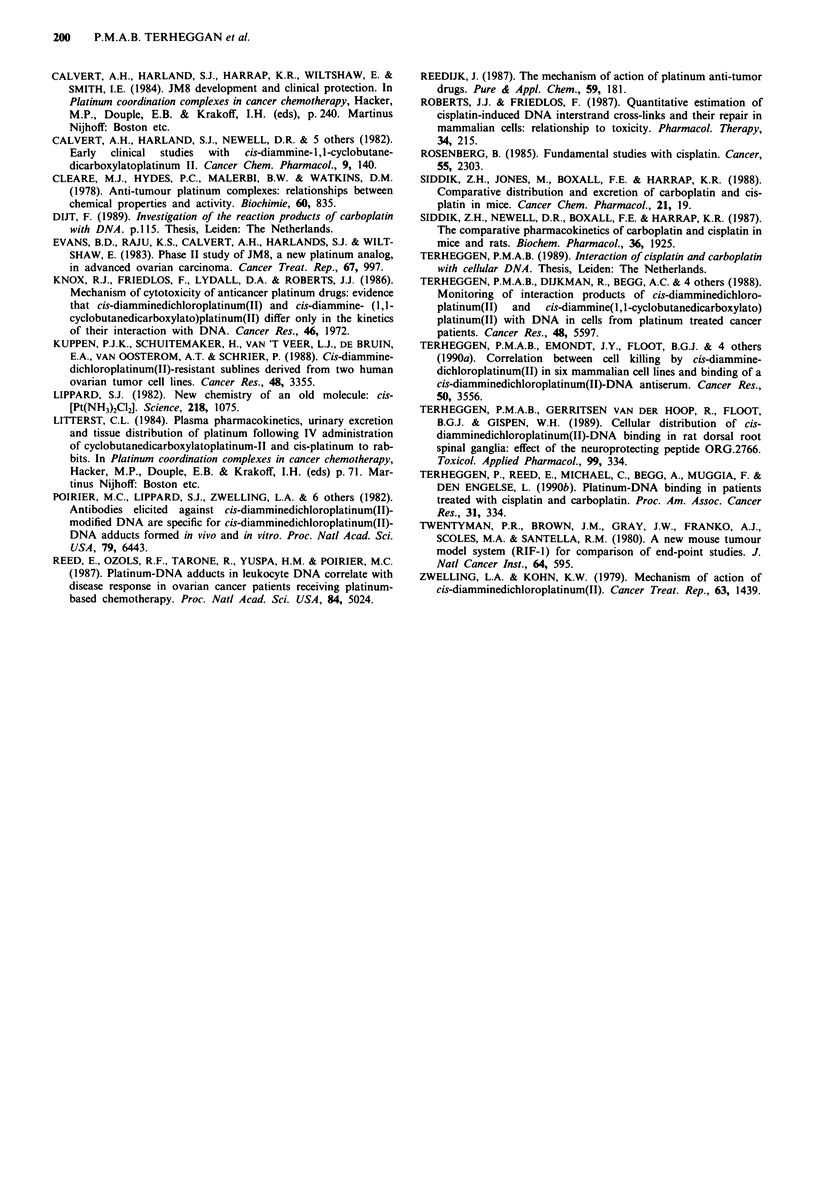# Formation of interaction products of carboplatin with DNA in vitro and in cancer patients.

**DOI:** 10.1038/bjc.1991.48

**Published:** 1991-02

**Authors:** P. M. Terheggen, A. C. Begg, J. Y. Emondt, R. Dubbelman, B. G. Floot, L. den Engelse

**Affiliations:** Division of Chemical Carcinogenesis, The Netherlands Cancer Institute (Antoni van Leeuwenhoek Huis), Amsterdam.

## Abstract

Binding of the cytostatic drug carboplatin to DNA was studied in solution, in RIF-1 and CHO cell lines and in human buccal cells after in vitro or in situ drug exposure. Results were compared with DNA adduction by cisplatin. The rate of binding in solution, determined by atomic absorption spectroscopy, was 35 times lower for carboplatin than for cisplatin. Adduct formation in cells in vitro was determined in a quantitative immunostaining assay. Staining intensities after carboplatin treatment were at least 29 times lower than after an equimolar dose of cisplatin. For RIF-1 and CHO cells, maximum levels of carboplatin-induced DNA modification were obtained 24 h after treatment; these levels correlated with cell killing. Adduct-specific staining in buccal cells from two carboplatin-treated patients increased 5-7 fold between 0 and 14 h after infusion, reaching a maximum at 10-14 h. This strongly contrasts with buccal cells from a cisplatin-treated patient, in which the adduct-specific staining signal increased by only 23% between 0 and 6 h after infusion, and then declined. This difference in the rate of adduct formation in vivo is consistent with the in vitro data.


Br. J. Cancer (1991), 63, 195-200 © Macmillan Press Ltd., 1991

### Formation of interaction products of carboplatin with DNA *in vitro* and in cancer patients

P.M.A.B. Terheggen[1,*], A.C. Begg[2], J.Y. Emondt[2], R. Dubbelman[3], B.G.J. Floot[1] & L. den Engelse[1]

[1]*Division of Chemical Carcinogenesis,* [2]*Division of Experimental Therapy, and* [3]*Clinical Research Unit, The Netherlands Cancer Institute (Antoni van Leeuwenhoek Huis), 121 Plesmanlaan, 1066 CX Amsterdam, The Netherlands.*

**Summary** Binding of the cytostatic drug carboplatin to DNA was studied in solution, in RIF-1 and CHO cell lines and in human buccal cells after *in vitro* or *in situ* drug exposure. Results were compared with DNA adduction by cisplatin. The rate of binding in solution, determined by atomic absorption spectroscopy, was 35 times lower for carboplatin than for cisplatin. Adduct formation in cells *in vitro* was determined in a quantitative immunostaining assay. Staining intensities after carboplatin treatment were at least 29 times lower than after an equimolar dose of cisplatin. For RIF-1 and CHO cells, maximum levels of carboplatin-induced DNA modification were obtained 24 h after treatment; these levels correlated with cell killing. Adduct-specific staining in buccal cells from two carboplatin-treated patients increased 5-7 fold between 0 and 14 h after infusion, reaching a maximum at 10-14 h. This strongly contrasts with buccal cells from a cisplatin-treated patient, in which the adduct-specific staining signal increased by only 23% between 0 and 6 h after infusion, and then declined. This difference in the rate of adduct formation *in vivo* is consistent with the *in vitro* data.

The drug *cis*-diamminedichloroplatinum(II) (*cis*DDP or cisplatin; Rosenberg, 1985) is widely used in the treatment of solid malignancies. *Cis*DDP is also an extremely toxic drug, with nephrotoxicity and neuropathy being the dose-limiting factors. To decrease these side effects, several platinum compounds have been synthesised and studied during the last decades. The second generation drug carboplatin (*cis*-diammine(1,1-cyclobutanedicarboxylato)platinum(II)) was found to be active against a wide spectrum of tumours, its nephrotoxicity is absent or low (Calvert *et al.*, 1984; Evans *et al.*, 1983), and its major toxic side effect is thrombocytopenia (Calvert *et al.*, 1982). The tissue distribution of carboplatin is almost the same as that of *cis*DDP (Siddik *et al.*, 1988). Carboplatin differs from *cis*DDP in its pharmacokinetics, i.e. the half-lives of free (ultrafilterable) platinum in blood plasma from both laboratory animals and humans are longer for carboplatin than for *cis*DDP (Litterst, 1984; Siddik *et al.*, 1987). The slow decrease of free carboplatin in plasma is in agreement with its slow and partially reversible *in vitro* binding to plasma proteins (Litterst, 1984; Siddik *et al.*, 1987). Differences in pharmacokinetics might explain why carboplatin can be active in patients with *cis*DDP-resistant tumours (Evans *et al.*, 1983; Calvert *et al.*, 1982). In the clinic, about four times higher chemotherapeutic doses of carboplatin are needed than *cis*DDP. This is consistent with the capacity to kill cells *in vitro*, which, for a given dose, is lower for carboplatin than for *cis*DDP (Bedford *et al.*, 1987; Kuppen *et al.*, 1988).

The interaction of carboplatin and *cis*DDP with DNA is considered to be important for their antitumour activity (Zwelling & Kohn, 1979; Lippard, 1982; Rosenberg, 1985; Reedijk, 1987). Evidence has been presented that carboplatin-DNA adducts, with the exception of mono-adducts, are chemically similar to *cis*DDP-DNA adducts (Knox *et al.*, 1986; Dijt, 1989). Because of the presumed importance of DNA adduct formation by platinum-containing drugs, we have previously studied *cis*DDP-DNA adducts in animal tissues, in cultured cells, and in cells from cancer patients using a quantitative immunoperoxidase method. The *cis*DDP-DNA adducts were assayed by an antiserum raised against *cis*DDP-modified DNA, which also recognised carboplatin-DNA adducts in a competitive ELISA and in a quantitative immunocytochemical peroxidase assay (Terheggen *et al.*, 1988, 1989).

Little is known on the formation of carboplatin-DNA adducts in cells or patients. Such knowledge might eventually contribute to the optimisation of patient anti-cancer treatment. We have therefore studied the binding of carboplatin to DNA in cultured cells and in buccal mucosa cells from carboplatin-treated cancer patients. The results were compared with adduct formation in DNA in solution and with the cytotoxic effects of carboplatin *in vitro*.

### Materials and methods

### DNA adduct formation in solution

One μmol calf thymus DNA-P in phosphate-buffer (0.2 mM $Na_2HPO_4$, 0.8 mM $NaH_2PO_4$, 3 mM NaCl; pH 7.4), pretreated with DNase $S_1$ (816 units $mg^{-1}$ DNA; Sigma, St. Louis, MO), was incubated with 0.4 μmol carboplatin (Carboplatin, Bristol-Myers, Weesp, The Netherlands) or 60 nmol *cis*DDP (Platinol, Bristol-Myers) for 0-72 h at 37°C in a volume of 1 ml. After 3-72 h, DNA was precipitated with ethanol overnight at − 20°C, washed twice with 70% aqueous ethanol to remove free platinum from DNA, and hydrolysed (Fichtinger-Schepman *et al.*, 1987). The platinum content of DNA samples was assessed with a Spectra Zeeman 30 flameless atomic absorption spectrometer (Varian, Zug, Switzerland).

### Cells and treatments

Murine RIF-1 cells (provided by Dr R.F. Kallman; Stanford University, CA) and CHO cells were grown as monolayer cultures in Ham's F10 medium (Flow Laboratories, Irvine, UK) + 10% foetal calf serum (FCS; Sera-Lab, Sussex, UK) under standard conditions (37°C in a humidified 5% $CO_2$:95% air mixture). RIF-1 cells were maintained involving alternating growth cycles *in vivo* and *in vitro* (Twentyman *et al.*, 1980). CHO cells were passaged weekly ten times before returning to a frozen stock. RIF-1 and CHO cells were seeded into 60 × 15 mm dishes and treated 2-4 days later when cells were in logarithmic growth phase. Freshly obtained buccal cells from volunteers ($10^3$-$10^4$ cells per person) were washed twice in RPMI 1640 medium prior to incubation with 75-300 μM carboplatin for 1-6 h or with 16.7 μM *cis*DDP for 1 h.

Correspondence: L. den Engelse.
*Present address: Institute of Molecular Medicine, John Radcliffe Hospital, Oxford, UK.
Parts of this paper have been included in the dissertation of P.M.A.B. Terheggen, University of Leiden, The Netherlands, 1989.
Received 28 March 1990; and in revised form 21 September 1990.

In a first series of experiments with RIF-1 and CHO, cells were treated for 2 h with carboplatin (0–4.7 mM) or *cis*DDP (5 μM, RIF-1; 16.7 μM, CHO), washed twice, trypsinised, and prepared for immunocytochemical analysis. In a second series of experiments, RIF-1 cells were incubated for 1 h with 1.2 mM (RIF-1) or 300 μM (CHO) carboplatin, washed twice, cultured in platinum-free medium for 0–48 h, and prepared for immunocytochemical analysis. In a third series of experiments, approximately $2 \times 10^5$ RIF-1 and CHO cells were treated for 1 h with 0–1.2 mM carboplatin or with 6.7 μM (RIF-1) or 33.3 μM (CHO) *cis*DDP, washed twice, cultured for 24 h in platinum-free medium, trypsinised, and assayed for their colony forming ability. Colony formation was assayed by culturing for 7–10 days. The surviving fraction was expressed as the plating efficiency relative to that of untreated cells. An aliquot of the cells was used for the immunocytochemical assay of platinum-DNA binding (Terheggen *et al.*, 1988). In a fourth series of experiments, approximately $10^8$ RIF-1 cells were incubated for 1 h with 1.2 mM carboplatin, washed twice, cultured for 0, 6 or 24 h in platinum-free medium, and harvested. Cytospin slides were made from an aliquot (approximately $10^4$ cells). DNA was isolated from the remaining cells essentially as described (Fichtinger-Schepman *et al.*, 1987) and hydrolysed in 1 M HCl for 1 h at 70°C. Platinum concentrations were assessed by atomic absorption spectroscopy (AAS).

### Human studies

The patients studied were treated at The Netherlands Cancer Institute. Patient A was treated for testicular cancer, patients B and C for ovarian cancer. Patients A and B were treated with 2 h infusions of 600 and 800 mg $m^{-2}$ carboplatin, respectively. Patient C received a 4 h infusion of 75 mg $m^{-2}$ *cis*DDP. These treatments were combined with either etoposide (500 mg $m^{-2}$, patient A) or cyclophosphamide (750 mg $m^{-2}$, patient C). The patients had been treated with *cis*DDP- or carboplatin-based chemotherapy 2 months (patient C), 3 months (patient A), or 10 months (patient B) earlier. Buccal cells were collected from patients (after informed consent) and healthy volunteers by wiping the inner side of the cheek with a cotton swab. Buccal cells from patients were collected immediately before infusion with *cis*DDP or carboplatin, and at between 0 and 48 h after the end of infusions. Cells were suspended in cold (0–4°C) RPMI 1640 medium (Gibco, Paisley, UK).

### Immunocytochemical assay

The preparation of antiserum NKI-A59 against *cis*DDP-modified DNA has been described previously (Terheggen *et al.*, 1988; Poirier *et al.*, 1982). NKI-A59 was used without purification at a dilution of 1:1800. The immunostaining assay was carried as described (Terheggen *et al.*, 1988). Slides from the same experiment were stained simultaneously and all samples were stained in two independent tests. The density of the adduct-specific nuclear staining was measured at the single-cell level as previously reported (Terheggen *et al.*, 1988). The nuclear staining density, expressed in arbitrary units, represents the integrated optical density per nucleus. In each slide, the nuclear staining density of 15 randomly selected nuclei was measured (two slides per point). To compare adduct levels of cells which had been incubated with different drug doses, and/or during different incubation times, the nuclear staining density was expressed per μM platinum per hour. The ratios of nuclear staining density $\mu M^{-1}$ drug, used for the comparison of carboplatin *vs* *cis*DDP within a certain cell type, were obtained from the average values of all available ratios of nuclear staining density $\mu M^{-1}$ drug.

### Statistics

Levels of significance were calculated using analysis of variance followed by Scheffé's test (Armitage, 1971). The best fitting line between points and the correlation coefficient, based on a 95% confidence interval, were calculated by linear regression analysis.

### Results

### Binding of carboplatin and cisDDP to DNA in solution

Carboplatin and *cis*DDP were reacted with calf thymus DNA for 0–72 h, free platinum was removed, and the platinum-DNA binding was measured by AAS. On a molar basis, 6.7 times more carboplatin than *cis*DDP was added to DNA. Figure 1 shows that the *cis*DDP-DNA binding reached a maximum between 6 and 24 h. In contrast, the reaction of carboplatin with DNA was still progressing after 24 h. The extent of carboplatin-DNA binding increased linearly with time (correlation coefficient >0.99, $P < 0.01$). The reaction rate of *cis*DDP with DNA, measured between 0 and 6 h, was 35 times higher than the reaction rate of carboplatin with DNA, measured between 0–72 h. The maximum platinum-nucleotide ratios were $5.8 \times 10^{-2}$ for *cis*DDP (mean from 20, 24 and 48 h) and $1.7 \times 10^{-2}$ for carboplatin (at 72 h).

**Figure 1** Binding of carboplatin and *cis*DDP to DNA in solution, expressed as platinum-nucleotide ratio, *vs* the incubation time (h). Carboplatin (0.4 μmol) or *cis*DDP (60 nmol) were incubated with 1 μmol DNA-P at 37°C in a total volume of 1 ml. At the indicated time points DNA was precipitated and analysed for platinum content by AAS.

### Platinum-DNA adducts in cultured cells directly after exposure

In these experiments, the initial carboplatin-DNA binding in cultured cells was determined and compared with *cis*DDP-DNA binding. RIF-1 and CHO cells were incubated for 2 h with carboplatin in concentrations up to 120 μM (RIF-1) or 4.72 mM (CHO). Chosen drug doses for the respective experiments were shown to give positive immunocytochemical staining and/or measurable levels of cell death. The nuclear staining density increased linearly with the dose (Figure 2; correlation coefficient >0.99, $P < 0.01$). The difference in nuclear staining density $\mu M^{-1}$ platinum between RIF-1 and CHO differed by up to a factor of 40. In the same experiment, RIF-1 and CHO cells were incubated for 2 h with *cis*DDP at concentrations of 5 μM for RIF-1 and 16.7 μM for CHO. The nuclear staining densities immediately after exposure were 201 and 190 arbitrary units for RIF-1 and CHO cells, respectively. The nuclear staining density $\mu M^{-1}$ *cis*DDP was therefore only 4.4 ± 0.1 fold higher for RIF-1 than for CHO cells, in contrast to the high value found for carboplatin. Comparing the two drugs within each cell line, a 64 ± 13 (for RIF-1 cells) and 527 ± 35 (for CHO cells) times higher molar concentration of carboplatin was required to achieve the same level of nuclear staining density as after *cis*DDP.

When buccal cells from healthy volunteers were incubated with carboplatin, the nuclear staining densities were approximately linear both with dose (correlation coefficient $>0.99$, $P<0.01$) and with incubation time (correlation coefficient $>0.97$, $P<0.05$; Figure 3). The nuclear staining density in these buccal cells (calculated from the data in Figure 3) ranged between 0.2 and 0.7 arbitrary units $\mu M^{-1}$ platinum $h^{-1}$. The corresponding values for RIF-1 were almost the same (0.2–0.5 arbitrary units $\mu M^{-1}$ platinum $h^{-1}$; calculated from Figure 4), but much lower figures were calculated for CHO cells (0.01–0.1 arbitrary units $\mu M^{-1}$ platinum $h^{-1}$; also from Figure 4). Buccal cells were also treated for 1 h with 16.7 µM *cis*DDP (results not shown). This revealed that, in agreement with the findings for RIF-1 and CHO cells, much more carboplatin than *cis*DDP was required to obtain the same nuclear staining density in buccal cells (i.e. by a factor 29 ± 5).

**Figure 2** Nuclear staining density (arbitrary units) *vs* the concentration of carboplatin. RIF-1 and CHO cells were treated for 2 h with carboplatin and immunostained for platinum-DNA binding. Each point represents the mean ± s.e. (bar) from two slides; 30 nuclei per point.

**Figure 3** Nuclear staining density of human buccal cells *vs* the concentration of carboplatin for different incubation times. Buccal cells from healthy volunteers were incubated with 75, 150, or 300 µM carboplatin for the times stated and immunostained for platinum-DNA binding. Each point represents the mean ± s.e. from two slides; 30 nuclei per point.

**Figure 4** Nuclear staining density *vs* time after exposure to carboplatin. Plateau-phase RIF-1 and CHO cells were incubated for 1 h with 300 µM (RIF-1) or 1.2 mM (CHO) carboplatin, washed, and cultured in platinum-free medium. At the indicated time points cells were immunostained for platinum-DNA binding. Each point represents the mean ± s.e. from two slides; 30 nuclei per point.

### *Carboplatin-DNA binding versus time after exposure*

To study the kinetics of carboplatin-DNA adduct formation, plateau-phase cells were incubated for 1 h with 300 µM (RIF-1) or 1.2 mM (CHO) carboplatin, washed, and cultured again in platinum-free medium for 0–48 h. These doses gave approximately equal adduct levels, as shown in pilot experiments. At 3 h, the nuclear staining density in CHO cells was still very low, whereas no adducts could be detected in RIF-1 cells (Figure 4). The nuclear staining density strongly increased between 6 and 24 h after exposure. Adduct levels were about the same in both cell lines after using a four times higher concentration of carboplatin for CHO cells. In another experiment (fourth series), RIF-1 cells were treated for 1 h with 1.2 mM carboplatin and harvested at different times thereafter. The nuclear staining densities at 0, 6 and 24 h post-treatment were 47 ± 26, 156 ± 21 and 287 ± 26, respectively, confirming the slow and progressive rise over this time period. The platinum-DNA binding, as measured by AAS, was undetectable at 0 h after exposure (i.e. platinum/nucleotide ratio $<10^{-5}$), while the platinum/nucleotide ratios were $1.7 \times 10^{-5}$ at 6 h, and $2.5 \times 10^{-5}$ at 24 h. This shows that, at 24 h, for each (arbitrary) immunostaining unit about 8,500 platinum molecules per nucleus ($10^{10}$ nucleotides) were present. It also indicates that the detection limit of AAS was higher (i.e. worse) than for immunocytochemistry. Since these AAS data were just above the detection limit it could not be definitely concluded that the nuclear staining densities paralleled total levels of platinum-DNA binding.

### *Survival and DNA adducts*

To investigate whether adduct levels parallel cell kill, the sensitivity of RIF-1 cells and CHO cells to carboplatin was related to the extent of carboplatin-DNA binding. Figure 5 shows the results from the clonogenic assay after a 1 h incubation with carboplatin in which cells were seeded 24 h after exposure. RIF-1 cells were approximately four times more sensitive to carboplatin than CHO cells. In this experiment, RIF-1 and CHO cells were also incubated for 1 h with 6.7 µM (RIF-1) or 33.3 µM (CHO) *cis*DDP, giving rise to surviving fractions of $1.8 \times 10^{-3}$ and $3.6 \pm 10^{-3}$, respectively. From these data it was estimated that a 35 times higher molar concentration of carboplatin than that of *cis*DDP was required to obtain the same toxic effects in these cell lines. The surviving fractions for cells plated immediately after treatment with carboplatin or 24 h later were not significantly different (results not shown).

Nuclear staining densities in cells taken from this experiment after a 24 h carboplatin-free incubation time are shown

**Figure 5** Survival of RIF-1 and CHO cells after treatment with carboplatin. Cells were treated for 1 h with 0–1.2 mM carboplatin, washed, cultured for 24 h in a platinum-free medium, and tested in a clonogenic assay. Each point represents the mean surviving fraction ± s.e. from two independent experiments.

**Figure 6** Formation of carboplatin-DNA adducts in RIF-1 and CHO cells. Cells were treated for 1 h with 0–1.2 mM carboplatin, washed, cultured for 24 h in carboplatin-free medium, and immunostained for platinum-DNA binding. Each point represents the mean ± s.e. from two slides; 30 nuclei per point.

in Figure 6. For CHO cells, a 4- to 5-fold higher concentration of carboplatin was required to reach the same level of nuclear staining density as for RIF-1 cells (Figure 6). This is in contrast to the high ratio found for cells measured immediately after exposure (Figure 2). The correlation coefficients between the nuclear staining density (at 24 h) and the surviving fractions were 0.95 for RIF-1 cells and 0.99 for CHO cells ($P < 0.01$). When data from both cell lines were combined, a good correlation remained (Figure 7; correlation coefficient: 0.89; $P < 0.01$). Comparison with data from the *cis*DDP-treated groups showed that 41–48 times more moles carboplatin than *cis*DDP were required to induce the same level of nuclear staining density. Although a slightly different regression line was obtained when data for *cis*DDP were pooled with those for carboplatin, the coefficient of correlation between nuclear staining density and surviving fraction remained the same. This was true for each of the cell lines and for the combined data of both lines (Figure 7). These experiments showed that carboplatin-DNA adduct levels in cultured cells correlated well with levels of cell killing. In addition, differences in cell survival between carboplatin and *cis*DDP were paralleled by differences in DNA adduct levels (Figure 7).

### *DNA adducts in cancer patients*

To test whether the time course of adduct formation found in DNA in solution and in cultured cells also applies to human cells *in situ*, three patients were investigated from which buccal cells were collected at different times after cisplatin or carboplatin therapy. Highest nuclear staining densities were measured 10 h (patient A) or 14 h (patient B) after the end of the infusions with carboplatin (Figure 8). The nuclear staining densities directly after the infusions did not differ significantly ($P < 0.05$) from pretreatment, i.e. background, values. The nuclear staining density increased about 9- (patient A) or 5-fold (patient B) between the end of the infusion and the time-point of highest adduct levels. The nuclear staining density increased significantly ($P < 0.01$)

**Figure 7** Correlation between the sensitivity to carboplatin and the carboplatin-DNA adduct-specific nuclear staining density. Survival data from Figure 5 are plotted against the nuclear staining densities from Figure 6. (RIF-1, ●; CHO, ○). Also shown are data for *cis*DDP-treated RIF-1 (▲) and CHO (△) cells. The best fitting line, calculated by linear regression analysis, concerns only the carboplatin data.

**Figure 8** Formation of carboplatin- and *cis*DDP-DNA adducts in buccal cells from cancer patients *vs* the time after the end of the infusions. Patients A (●) and B (▲) received 600 and 800 mg m$^{-2}$ carboplatin, respectively, via a 2 h-infusion. Patient C (◆) received 75 mg m$^{-2}$ *cis*DDP by a 230 min-infusion. Cells were washed, fixed, and immunostained for platinum-DNA adducts. Each point represents the mean ± s.e. from two slides; 30 nuclei per point.

between 6 and 10 h (patient A) and between 6 and 14 h (patient B). The observed maximum nuclear staining density $\mu mol^{-1}$ carboplatin in buccal cells from patient B was 1.4 times higher than in those from patient A. Although the number of time-points was relatively small for practical reasons, the results suggest some interpatient variation. The nuclear staining density in buccal cells from the *cis*DDP-treated patient C showed no significant incease ($P > 0.05$) between 0 and 6 h after the end of the infusion (Figure 8), showing that the level of *cis*DDP-DNA adducts was almost maximal immediately after infusion. From the data shown in Figure 8 it was calculated that a six times molar excess of carboplatin over *cis*DDP was required to achieve the same nuclear staining density in human buccal cells *in situ*.

### Discussion

Carboplatin loses its cyclobutanedicarboxylato acid-ligand when it bifunctionally binds to DNA. The ultimate interaction products of carboplatin with DNA are, therefore probably chemically identical with those of *cis*DDP (Knox *et al*., 1986; Dijt, 1989). This could explain why antiserum NKI-A59 raised against *cis*DDP-modified DNA, also recognises carboplatin-DNA adducts (Terheggen *et al*., 1988; compare also Reed *et al*., 1987).

The binding of carboplatin to DNA in solution was shown to proceed slower than that of *cis*DDP to DNA (Figure 1). It is known that only aquated derivatives of *cis*DDP and carboplatin can bind to DNA (Zwelling & Kohn, 1979). Carboplatin's lower interaction rate with DNA can therefore be explained by its aquation rate, which can be up to 100 times lower than that of *cis*DDP (Cleare *et al*., 1978; Knox *et al*., 1986). The relatively low aquation rate of carboplatin also explains why relatively high concentrations of this drug are required to obtain detectable adduct levels in cultured cells (Figures 2 and 3). An additional explanation for the relatively low adduct-specific immunostaining signals in the latter experiments follows from Figure 4. This figure shows a substantial increase in the adduct-specific immunosignal of both CHO and RIF-1 cells after removal of carboplatin from the incubation medium. Although it cannot be excluded that these cells retain significant amounts of unreacted carboplatin after being washed twice, a more reasonable explanation seems to be the post-treatment conversion of already formed monofunctional carboplatin-DNA adducts into bidentate adducts (Knox *et al*., 1986). Due to their bulky cyclobutanedicarboxylato acid group, the monovalent carboplatin-DNA adducts might not be recognised by antiserum NKI-A59, whereas the ultimate bidentate adducts, identical to bidentate *cis*DDP-DNA adducts, will be recognised. The relative contributions of these two factors, aquation rate and adduct recognition, to the low initial staining after carboplatin is yet unknown.

Our findings in cultured cells are in agreement with those reported in the literature. For example, carboplatin-DNA adduct levels in cultured Walker and V79 cells were found to be up to 40 times lower at a molar basis than *cis*DDP-DNA adduct levels (Knox *et al*., 1986). The slow binding of carboplatin to DNA in solution (Figure 1) and, apparently, also to DNA in cultured cells (Figure 4) is consistent with published data on DNA-DNA cross-linking in cultured cells, which is maximal at 18 h post-treatment for carboplatin and between 6 and 12 h for *cis*DDP (Roberts & Friedlos, 1987). These data are reminiscent of our present observation that the *in situ* rate of platinum-DNA binding in buccal cells from patients was markedly slower for carboplatin than for *cis*DDP (Figure 8). Other *in vivo* data (on rat kidneys) also suggest that carboplatin binds slower and to a much lower extent to cellular DNA than does *cis*DDP (Terheggen, 1989). However, it is emphasised that these *in vivo* data cannot be compared directly with *in vitro* results (see below). In the present study it was shown that the immunostaining signal paralleled killing of cultured RIF-1 and CHO cells when both parameters were measured at 24 h after treatment (Figure 7). In fact, a 4-fold difference between the two cell lines was found for both sensitivity to carboplatin and nuclear staining density. A similar correlation has previously been demonstrated for six *cis*DDP-treated mammalian cell lines (Terheggen *et al*., 1990*a*). A comparison of Figure 2 with Figure 6 shows that the difference in nuclear staining density between RIF-1 and CHO cells was up to a factor 40 directly after treatment with carboplatin, and only a factor 4 to 5 at 24 h. This discrepancy suggests that the rate of net DNA adduction and/or the conversion rate of monovalent into bidentate adducts in one (or both) of these cells is not constant in time. Further investigations are needed to confirm the observed difference and to discriminate between the two possible explanations.

There was a marked difference between the *in vitro* and *in vivo* data on DNA modification by carboplatin. When highest levels of carboplatin-DNA binding were compared with the highest levels of *cis*DDP-DNA adducts, 41–48 times more moles carboplatin than *cis*DDP were required to reach a given level of platinum-DNA binding in cultured RIF-1 and CHO cells (see Results). This factor was only 6 to 17 when buccal cells from carboplatin- and *cis*DDP-treated cancer patients were compared (Figure 8; see Terheggen *et al*., 1988) and 4.3 ± 1.3 (mean ± s.e.) for buccal cells from 11 patients treated with carboplatin and *cis*DDP on different days (Terheggen *et al*., 1990*b*; study in cooperation with F.M. Muggia, M.D., University of Southern California). An explanation for this *in vitro*/*in vivo* difference could be as follows. A lower binding level of carboplatin to DNA relative to *cis*DDP may partially be due to a decreased drug uptake. A prolonged drug exposure, as occurred in the patients, would allow more time for the uptake of carboplatin. The prolonged availability of free carboplatin in the blood plasma compared with that for free *cis*DDP (Calvert *et al*., 1984) would therefore contribute to the difference between *in vivo* and *in vitro* results.

A high correlation was shown between cell killing and the level of platinum-DNA binding, when measured 24 h after incubation with carboplatin (this paper) or directly after exposure to *cis*DDP (Terheggen *et al*., 1990*a*). This suggests that, irrespective of the cell type or the drug, the adduct-specific immunostaining signal is a quantitative indicator for *in vitro* cell kill, provided that time points of maximum DNA adduction are studied. The latter condition might be important especially for slowly reacting drugs like carboplatin. The cell kill/adduct correlation and the ability of the antiserum to detect carboplatin adducts at clinically relevant doses, coupled with the knowledge of the optimal measuring time, opens the possibility of immuno-staining tumour biopsy material for drug sensitivity.

This study was supported by grant NKI 86-11 from the Dutch Cancer Society (Koningin Wilhelmina Fonds). The authors wish to thank C. Michael and O. van Tellingen for technical assistance, Dr G. Los for his cooperation in the rat experiments and helpful discussions and Dr S. Rodenhuis for critical reading of the manuscript.

### References

ARMITAGE, P. (1971). *Statistical Methods in Medical Research* pp. 202–207. Blackwell: Oxford.

BEDFORD, P., SHELLARD, S.A., WALKER, M.C., WHELAN, R.D.H., MASTERS, J.R.W. & HILL, B.T. (1987). Differential expression of collateral sensitivity of resistance to cisplatin in human bladder carcinoma cell lines pre-exposed *in vitro* to either X-irradiation or cisplatin. *Int. J. Cancer*, **40**, 681.

CALVERT, A.H., HARLAND, S.J., HARRAP, K.R., WILTSHAW, E. & SMITH, I.E. (1984). JM8 development and clinical protection. In *Platinum coordination complexes in cancer chemotherapy*, Hacker, M.P., Douple, E.B. & Krakoff,. I.H. (eds), p. 240. Martinus Nijhoff: Boston etc.

CALVERT, A.H., HARLAND, S.J., HARRAP, K.R., WILTSHAW, E. & SMITH, I.E. (1984). JM8 development and clinical protection. In *Platinum coordination complexes in cancer chemotherapy*, Hacker, M.P., Douple, E.B. & Krakoff, I.H. (eds), p. 240. Martinus Nijhoff: Boston etc.

CALVERT, A.H., HARLAND, S.J., NEWELL, D.R. & 5 others (1982). Early clinical studies with *cis*-diammine-1,1-cyclobutane-dicarboxylatoplatinum II. *Cancer Chem. Pharmacol.*, **9**, 140.

CLEARE, M.J., HYDES, P.C., MALERBI, B.W. & WATKINS, D.M. (1978). Anti-tumour platinum complexes: relationships between chemical properties and activity. *Biochimie*, **60**, 835.

DIJT, F. (1989). *Investigation of the reaction products of carboplatin with DNA*. p.115. Thesis, Leiden: The Netherlands.

EVANS, B.D., RAJU, K.S., CALVERT, A.H., HARLANDS, S.J. & WILTSHAW, E. (1983). Phase II study of JM8, a new platinum analog, in advanced ovarian carcinoma. *Cancer Treat. Rep.*, **67**, 997.

KNOX, R.J., FRIEDLOS, F., LYDALL, D.A. & ROBERTS, J.J. (1986). Mechanism of cytotoxicity of anticancer platinum drugs: evidence that *cis*-diamminedichloroplatinum(II) and *cis*-diammine- (1,1-cyclobutanedicarboxylato)platinum(II) differ only in the kinetics of their interaction with DNA. *Cancer Res.*, **46**, 1972.

KUPPEN, P.J.K., SCHUITEMAKER, H., VAN 'T VEER, L.J., DE BRUIN, E.A., VAN OOSTEROM, A.T. & SCHRIER, P. (1988). *Cis*-diammine-dichloroplatinum(II)-resistant sublines derived from two human ovarian tumor cell lines. *Cancer Res.*, **48**, 3355.

LIPPARD, S.J. (1982). New chemistry of an old molecule: *cis*-$[Pt(NH_3)_2Cl_2]$. *Science*, **218**, 1075.

LITTERST, C.L. (1984). Plasma pharmacokinetics, urinary excretion and tissue distribution of platinum following IV administration of cyclobutanedicarboxylatoplatinum-II and cis-platinum to rabbits. In *Platinum coordination complexes in cancer chemotherapy*, Hacker, M.P., Douple, E.B. & Krakoff, I.H. (eds) p. 71. Martinus Nijhoff: Boston etc.

POIRIER, M.C., LIPPARD, S.J., ZWELLING, L.A. & 6 others (1982). Antibodies elicited against *cis*-diamminedichloroplatinum(II)-modified DNA are specific for *cis*-diamminedichloroplatinum(II)-DNA adducts formed *in vivo* and *in vitro*. *Proc. Natl Acad. Sci. USA*, **79**, 6443.

REED, E., OZOLS, R.F., TARONE, R., YUSPA, H.M. & POIRIER, M.C. (1987). Platinum-DNA adducts in leukocyte DNA correlate with disease response in ovarian cancer patients receiving platinum-based chemotherapy. *Proc. Natl Acad. Sci. USA*, **84**, 5024.

REEDIJK, J. (1987). The mechanism of action of platinum anti-tumor drugs. *Pure & Appl. Chem.*, **59**, 181.

ROBERTS, J.J. & FRIEDLOS, F. (1987). Quantitative estimation of cisplatin-induced DNA interstrand cross-links and their repair in mammalian cells: relationship to toxicity. *Pharmacol. Therapy*, **34**, 215.

ROSENBERG, B. (1985). Fundamental studies with cisplatin. *Cancer*, **55**, 2303.

SIDDIK, Z.H., JONES, M., BOXALL, F.E. & HARRAP, K.R. (1988). Comparative distribution and excretion of carboplatin and cisplatin in mice. *Cancer Chem. Pharmacol.*, **21**, 19.

SIDDIK, Z.H., NEWELL, D.R., BOXALL, F.E. & HARRAP, K.R. (1987). The comparative pharmacokinetics of carboplatin and cisplatin in mice and rats. *Biochem. Pharmacol.*, **36**, 1925.

TERHEGGEN, P.M.A.B. (1989). *Interaction of cisplatin and carboplatin with cellular DNA*. Thesis, Leiden: The Netherlands.

TERHEGGEN, P.M.A.B., DIJKMAN, R., BEGG, A.C. & 4 others (1988). Monitoring of interaction products of *cis*-diamminedichloro-platinum(II) and *cis*-diammine(1,1-cyclobutanedicarboxylato) platinum(II) with DNA in cells from platinum treated cancer patients. *Cancer Res.*, **48**, 5597.

TERHEGGEN, P.M.A.B., EMONDT, J.Y., FLOOT, B.G.J. & 4 others (1990*a*). Correlation between cell killing by *cis*-diammine-dichloroplatinum(II) in six mammalian cell lines and binding of a *cis*-diamminedichloroplatinum(II)-DNA antiserum. *Cancer Res.*, **50**, 3556.

TERHEGGEN, P.M.A.B., GERRITSEN VAN DER HOOP, R., FLOOT, B.G.J. & GISPEN, W.H. (1989). Cellular distribution of *cis*-diamminedichloroplatinum(II)-DNA binding in rat dorsal root spinal ganglia: effect of the neuroprotecting peptide ORG.2766. *Toxicol. Applied Pharmacol.*, **99**, 334.

TERHEGGEN, P., REED, E., MICHAEL, C., BEGG, A., MUGGIA, F. & DEN ENGELSE, L. (1990*b*). Platinum-DNA binding in patients treated with cisplatin and carboplatin. *Proc. Am. Assoc. Cancer Res.*, **31**, 334.

TWENTYMAN, P.R., BROWN, J.M., GRAY, J.W., FRANKO, A.J., SCOLES, M.A. & SANTELLA, R.M. (1980). A new mouse tumour model system (RIF-1) for comparison of end-point studies. *J. Natl Cancer Inst.*, **64**, 595.

ZWELLING, L.A. & KOHN, K.W. (1979). Mechanism of action of *cis*-diamminedichloroplatinum(II). *Cancer Treat. Rep.*, **63**, 1439.